# Macrophage numbers in the marginal area of sarcomas predict clinical prognosis

**DOI:** 10.1038/s41598-023-28024-1

**Published:** 2023-01-23

**Authors:** Michinobu Umakoshi, Akiko Nakamura, Hiroyuki Tsuchie, Zhuo Li, Yukitsugu Kudo-Asabe, Ken Miyabe, Yukinobu Ito, Makoto Yoshida, Hiroyuki Nagasawa, Kyoji Okada, Hiroshi Nanjo, Daichi Maeda, Naohisa Miyakoshi, Masamitsu Tanaka, Akiteru Goto

**Affiliations:** 1 Department of Cellular and Organ Pathology, Graduate School of Medicine, Akita University, 1-1-1 Hondo, Akita, 010-8543 Japan; 2 Department of Orthopedic Surgery, Graduate School of Medicine, Akita University, Akita, Japan; 3 Department of Laboratory Medicine, The First Affiliated Hospital of Xi’an Medical University, Xi’an, 710077 China; 4 Department of Physical Therapy, Graduate School of Health Science, Akita University, Akita, Japan; 5 Department of Pathology, Akita University Hospital, Akita, Japan; 6 Department of Molecular and Cellular Pathology, Graduate School of Medical Sciences, Kanazawa University, Kanazawa, Japan; 7 Department of Molecular Medicine and Biochemistry, Graduate School of Medicine, Akita University, Akita, Japan

**Keywords:** Cancer, Diseases, Oncology, Risk factors

## Abstract

Even when treated comprehensively by surgery, chemotherapy, and radiotherapy, soft-tissue sarcoma has an unfavorable outcome. Because soft-tissue sarcoma is rare, it is the subject of fewer clinicopathological studies, which are important for clarifying pathophysiology. Here, we examined tumor-associated macrophages in the intratumoral and marginal areas of sarcomas to increase our knowledge about the pathophysiology. Seventy-five sarcoma specimens (not limited to a single histological type), resected at our institution, were collected, and the number of CD68-, CD163-, and CD204-positive macrophages in the intratumoral and marginal areas was counted. We then performed statistical analysis to examine links between macrophage numbers, clinical factors, and outcomes. A high number of macrophages positive for all markers in both areas was associated with worse disease-free survival (DFS). Next, we divided cases according to the FNCLCC classification (Grade 1 and Grades 2/3). In the Grade 1 group, there was no significant association between macrophage number and DFS. However, in the Grade 2/3 group, high numbers of CD163- and CD204-positive macrophages in the marginal area were associated with poor DFS. By contrast, there was no significant difference between the groups with respect to high or low numbers of CD68-, CD163-, or CD204-positive macrophages in the intratumoral area. Multivariate analysis identified the number of CD163- and CD204-positive macrophages in the marginal area as an independent prognostic factor. Macrophage numbers in the marginal area of soft-tissue sarcoma may better reflect clinical behavior.

## Introduction

Soft-tissue sarcoma is relatively rare compared with other cancers; therefore, there are problems related to diagnosis and treatment. Generally, although high-grade soft-tissue sarcomas can be managed by comprehensive treatment (e.g., surgery, chemotherapy, and radiotherapy), they often recur. Thus, our knowledge about tumorigenesis and progression must improve if we are to obtain better treatment outcomes. Soft-tissue sarcomas are differentiated into various tissues with different clinicopathological features and genomic status. This makes clarifying the pathophysiology complicated. Here, we focus on the tumor microenvironment of sarcoma, rather than the sarcoma itself.

The tumor microenvironment comprises various tissues and cell types, including immune cells (e.g., macrophages), endothelial cells, fibroblasts, and adipocytes. Recently, the role of tumor-associated macrophages (TAMs), which contribute to development and progression of cancer, has received much attention. Upon stimulation with various factors, macrophages are induced to differentiate into an M1 or an M2 phenotype, each of which plays a different role. Toll-like receptor ligands, lipopolysaccharide, IFN-γ, and GM-CSF induce M1 macrophages (referred to as classically activated macrophages), which (generally) play a pro-inflammatory/antitumor role^1–3^. By contrast, cancer cells and the tumor microenvironment, along with IL4, IL10, IL13, transforming growth factor beta (TGF), and prostaglandin E2 (PGE2), induce M2 macrophages or TAMs, which promote tumor progression by secreting growth factors, adhesion factors, and cytokines^1–6^. M1 macrophages and TAMs (M2) can be distinguished by immunophenotyping. Typically, CD68 and Iba1 are pan-macrophage markers, whereas CD163 and CD204 are TAM-specific markers^7–9^. Many studies report that TAMs play a pro-tumorigenic role by forming a cancer-promoting inflammatory microenvironment^10–12^; they do this by exerting immunosuppressive effects^12–14^, and by promoting angiogenesis^15,16^ and metastasis^17–19^. In fact, many clinicopathological studies of various cancers show that aggressive invasion by macrophages is associated with a poor prognosis^20–27^. By contrast, fewer studies have investigated the clinicopathological significance of macrophages present in sarcomas.

Previously, we examined the role of TAMs and found that they transfer cancer-derived components to stromal cells to establish a pro-tumorigenic microenvironment^28^. Fibroblasts exposed to cancer-derived components differentiate into cancer-associated fibroblast (CAF)-like cells in the marginal area, which then form a pro-tumoral microenvironment. We hypothesized that macrophages play a role in the transfer of cancer-derived components to marginal stromal cells, and that this contributes to enlargement of the tumor microenvironment. Accordingly, macrophages in the marginal area may play a role equally important to that of macrophages in the intratumoral area, and this may also be true for sarcomas. In this study, we subjected surgical specimens of 75 soft-tissue sarcomas to immunohistochemical examination and counted the number of the infiltrating CD68-positive macrophages (total macrophages) and CD163- and CD204-positive macrophages (TAMs). We counted each type in the intratumoral area and marginal area (the non-sarcoma area adjacent to the sarcoma) and examined the association between macrophage numbers and various clinical factors.

## Materials and methods

### Case collection

Seventy-five cases of soft-tissue sarcoma, evaluated according to the FNCLCC grading system, were enrolled. These cases were managed at Akita University Hospital (Akita, Japan) between 2004 and 2017. The general characteristics of the 75 patients are shown in Table 1. No patients received preoperative chemotherapy or radiotherapy, and all underwent marginal or extended resection. The clinicopathological characteristics were obtained from clinical records; however, some cases were re-diagnosed according to the current WHO pathological classification (5th edition) due to changes in classification, terms, and criteria over the last 20 years. Collected histological subtypes included well-differentiated liposarcoma, myxoid liposarcoma, round cell liposarcoma, dedifferentiated liposarcoma, pleomorphic liposarcoma, myxofibrosarcoma, conventional leiomyosarcoma, poorly differentiated leiomyosarcoma, synovial sarcoma, extraskeletal myxoid chondrosarcoma, and undifferentiated pleomorphic sarcoma. Disease-free survival (DFS) was measured from the date of surgery to the date of recurrence or the date when the patient was last known to be disease-free. Ethical approval was obtained from the Ethics Committee of Akita University, Graduate School of Medicine (Reference No. 2652). Informed consent was obtained in the form of opt-out on the website. This study was performed in accordance with the Declaration of Helsinki.

**Table 1:** Details of all enrolled cases.

Total	75
Sex	
Male	38
Female	37
Age	64.5 ± 16.3
< 65	31
> = 65	44
Tumor size	11.1 ± 5.42
< = 5 cm	7
> 5 cm to 10 cm > =	31
> 10 cm	37
Location	
Extremities	52
Others	23
Histology	
Well-differentiated liposarcoma	11
Myxoid liposarcoma	9
Round cell liposarcoma	1
Dedifferentiated liposarcoma	11
Pleomorphic liposarcoma	6
Myxofibrosarcoma	15
Conventional leiomyosarcoma	3
Poorly differentiated leiomyosarcoma	1
Synovial sarcoma	3
Extraskeletal myxoid chondrosarcoma	1
Undifferentiated pleomorphic sarcoma	14
FNCLCC	
Grade 1	31
Grade 2	22
Grade 3	22

### Immunohistochemistry (IHC)

Tissue sections (thickness: 4 μm) were cut and stained by a Ventana Discovery XT autostainer (Ventana Medical Systems, Tucson, AZ, USA). The staining antibodies were: mouse monoclonal anti-human CD68 (Clone PG-M1, 1:100; Dako, Japan), a pan-macrophage marker, anti-mouse/rat /human CD163 (Clone EPR19518, 1:500, abcam, Cambridge, UK), and anti-human CD204 (Clone SRA-E5, 1:500; Trans Genic Inc., Japan), a TAM marker.

### IHC evaluation

**Figure 1:**
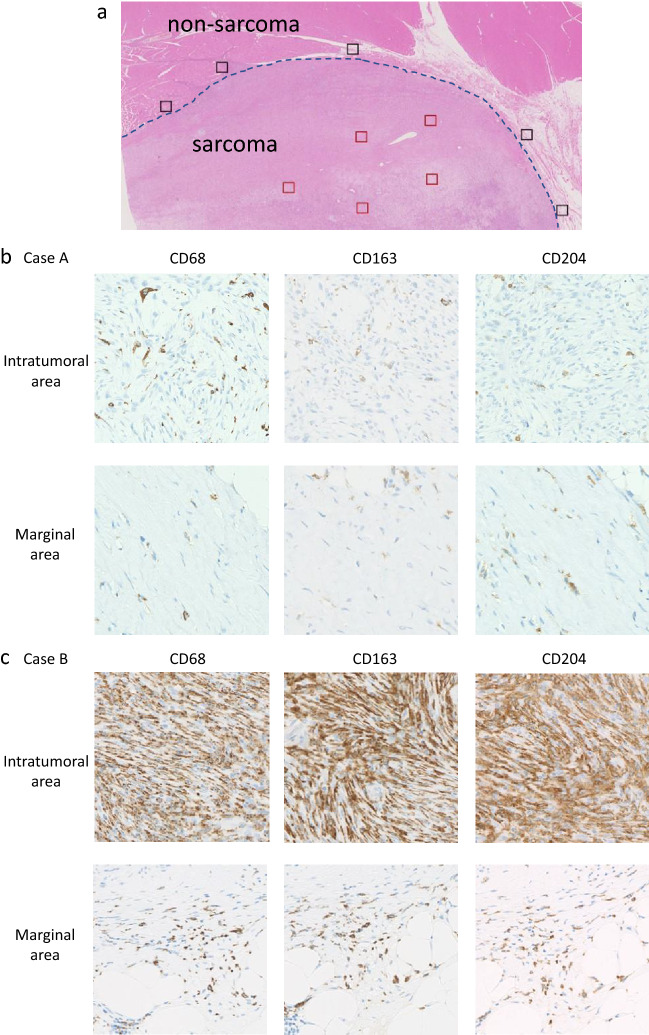
A representative case demonstrating the areas that were counted. Red square, intratumoral area; black square, marginal area (**a**). Representative immunohistochemical images of the intratumoral and marginal areas in case A (FNCLCC Grade 1 sarcoma) (**b**), and case B (FNCLL Grade 3 sarcoma) (**c**).

All IHC slides were scanned at an absolute magnification of 20 × using a pathology digital imaging system (Nanozoomer virtual slide system, Hamamatsu Photonics, Shizuoka, Japan). For each slide, five representative fields (0.2 mm^2^) containing intratumoral and marginal areas were selected, and the number of positive cells was counted manually (Fig. 1a). The selected intratumoral and marginal areas were almost same for the CD68, CD163, and CD204-stained slides. The marginal area is the non-sarcoma area adjacent to the sarcoma. For most sarcomas, the histological findings identified a non-sarcomatous area bordering the sarcomatous area, which was designated as the “marginal area”. In cases where the border between sarcoma and non-sarcoma was unclear, we carefully distinguished atypical cells from preexisting tissue, and designated the non-sarcoma area around the sarcoma margin as the “marginal area”. The total number of CD68-, CD163-, or CD204-positive cells in the five tumor fields was recorded (Fig. 1b,c). There were two cases in which the marginal specimen did not include normal tissue; in these cases, the marginal tumor area was used instead of the marginal normal area. Both cases involved an atypical lipomatous tumor.

### Statistical analysis

**Figure 2:**
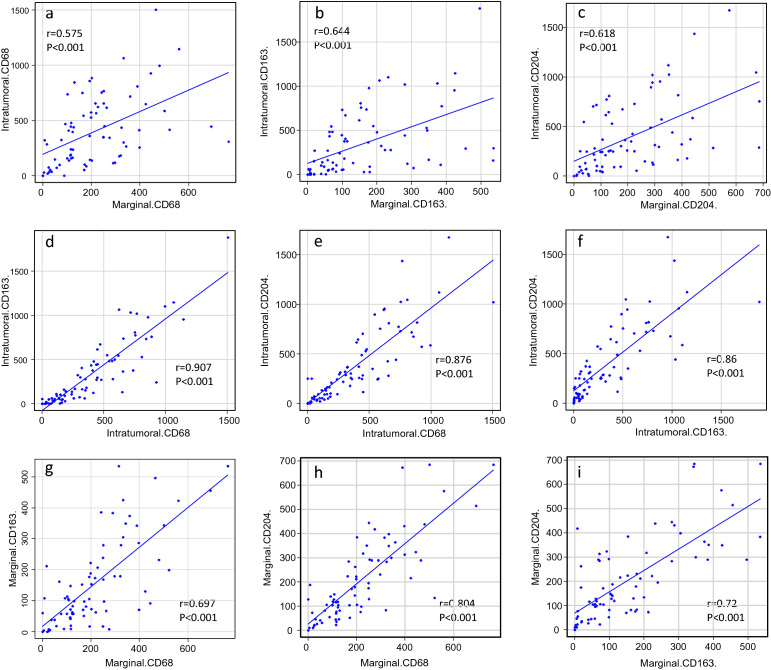
Correlation between the number of each marker-positive macrophage in the intratumoral area and that in the marginal area.

**Figure 3:**
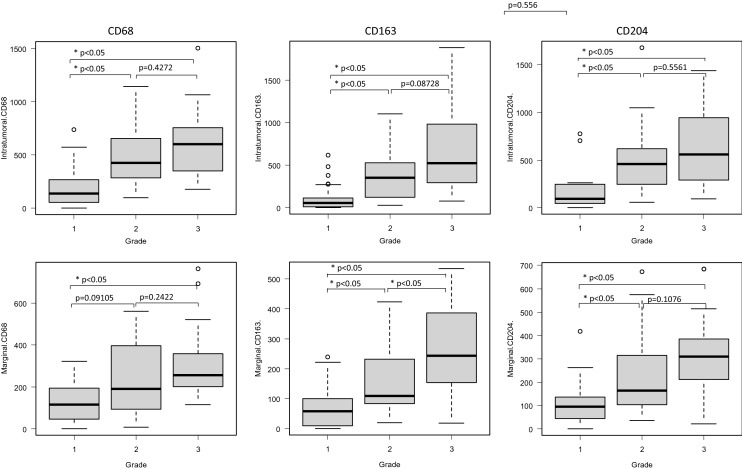
Comparison of the average numbers of macrophages according to FNCLCC grade. *Kruskal–Wallis test. Post-hoc test = Steel–Dwass test.

Shapiro–Wilk test showed the number of macrophages are not normal distribution, therefore, the non-parametric analyses described below were performed. The number, distribution, and type of macrophage was assessed using the Friedmann rank sum test with Bonferroni correction, and the Wilcoxon signed rank test (Table 2). The correlation between the number of intratumoral/marginal macrophages of each phenotype was assessed using Spearman’s rank correlation analysis (Fig. 2). Clinical factors and macrophage type were assessed using the Mann–Whitney U test, the Kruskal–Wallis test, and the Steel–Dwass test (Table 3, Fig. 3). The log-rank test and a Cox proportional hazards regression model were also used. All statistical analyses were performed using EZR (Saitama Medical Centre, Jichi Medical University), which is based on R (The R Foundation for Statistical Computing, Vienna, Austria, version 4.1.2), and R commander^29^. *P* values of < 0.05 were considered significant. Correlation coefficients between 0.4 and 0.7 were defined as intermediate, whereas those > 0.7 were defined as strong.

**Table 2:** Number of CD68-, CD163-, or CD204-positive macrophages in the intratumoral and marginal areas.

	Intratumoral area	Marginal area	P-value
CD68-positive macrophages	349(147–602.5)	193(107–306)	0.00000109**
CD163-positive macrophages	180(59.5–491.5)	106(58–217)	0.000013**
CD204-positive macrophages	260(99.5–603)	145(80.5–296.5)	0.00000711**
P-value	0.000233*	0.00000283*	

All parameters are expressed as the median, and 25th and 75th centiles.

*Friedman rank sum test (with post-hoc Bonferroni correction).

**Wilcoxon signed rank test.

**Table 3:** Number of macrophages in the intratumoral and marginal areas, and their association with clinical factors.

	n	Intra-tumoral CD68 +	P value	Marginal CD68 +	P value	Intra-tumoral CD163 +	P value	Marginal CD163 +	P value	Intra-tumoral CD204 +	P value	Marginal CD204 +	P value
Sex													
Male	38	312 (150.25–582.5)	0.924	181.5 (103.25–316.5)	0.66	219.5 (61.25–521.5)	0.719	103.5 (49.75–261.75)	0.983	265.5 (95.5–627.5)	0.743	147.5 (48.5–310.5)	0.494
Female	37	350 (146–617)		194 (108–289)		180 (58–435)		106 (59–535)		260 (135–587)		145 (106–290)	
Age													
< 65	31	237 (128.5–464.5)	0.0986	198 (114.5–318)	0.583	111 (58.5–278.5)	0.0758	98 (52.5–230.5)	0.914	245 (85.5–359)	0.0666	196 (80.5–303.5)	0.91
≥ 65	44	426.5 (185.5–654.75)		174.5 (104.75–265)		370 (61–630)		109 (58.75–208.25)		318.5 (160–723)		137.5 (81.25–292.5)	
Tumor size													
≤ 5 cm	7	410 (354.5–493.5)	0.858	199 (149.5–323)	0.864	180 (128–278.5)	0.955	131 (106–230.5)	0.319	260 (252.5–401)	0.825	196 (137–220)	0.329
≥ 5.1 – ≤ 10 cm	31	416 (141.5–635.5)		179 (108–256)		272 (56.5–522)		83 (55–175.5)		259 (116–630)		106 (79.5–243)	
> 10 cm	37	303 (148–545)		201 (108–332)		160 (58–529)		109 (47–342)		285 (92–619)		188 (55–351)	
Location													
Extremities	52	346.5 (143.75–618.5)	0.761	192.5 (104.75–279.75)	0.722	173.5 (60.25–494.5)	0.792	101 (55.25–184)	0.298	256 (112.25–626)	0.818	142.5 (83–294.75)	0.886
Other	23	350 (171–566.5)		198 (115.5–322)		272 (81–491.5)		174 (65–283.5)		304 (99.5–567)		174 (75–319.5)	
FNCLCC													
Grade 1	31	137 (53.5–268)	< 0.05*	115 (46.5–193.5)	< 0.05*	56 (11–115.5)	< 0.05*	58 (9–100.5)	< 0.05*	95 (43.5–247.5)	< 0.05*	96 (45.5–136.5)	< 0.05*
Grade 2	22	425.5 (295.5–655)		190 (96.25–395)		351 (125–508.25)		109 (85.25–227)		458 (257.5–611)		164 (104.5–308.75)	
Grade 3	22	602.5 (366.5–754)		256 (204.75–355)		522 (297.5–938.5)		243.5 (159–383)		557.5 (293.5–932.75)		309 (215–384.75)	

The number of intratumoral CD68 + , CD163 + , or CD204 + and marginal CD68 + , CD163 + , or CD204 + cells represent the average number of each phenotype in the intratumoral and marginal area.

FNCLCC: Fédération Nationale des Centres de Lutte Contre le Cancer.

*Kruskal–Wallis test.

### Ethical approval

Ethical approval was obtained from the Akita University, Faculty of Medicine, Ethics Committee (Reference No. 2652).

### Patient consent status

Informed consent was obtained in the form of opt-out on the website.

## Results

### Distribution of each macrophage phenotype, and the correlation between intratumoral and marginal macrophages of each phenotype in soft-tissue sarcoma

The number of CD68-, CD163-, and CD204-positive macrophages in the intratumoral and marginal areas of 75 soft-tissue sarcoma specimens was counted. A comparison of macrophage numbers in the intratumoral and marginal areas revealed that the number expressing each of these markers was significantly higher in the intratumoral area than in the marginal area (CD68-, CD163-, and CD204-positive macrophages: p = 0.00000109, p = 0.000013, and p = 0.00000711, respectively) (Table 2). For each particular marker, there was an intermediate correlation between the numbers in the marginal and intratumoral areas (CD68-positive macrophages: r = 0.575; CD163-positive macrophages: r = 0.644; CD204-positive macrophages: r = 0.618; p < 0.001 for all) (Fig. 2a–c).

By contrast, a comparison of the number of macrophages expressing each type of marker revealed that the number of CD163-positive macrophages in both areas was significantly lower than that of CD68- and CD204-positive macrophages (intratumoral area: p = 0.000233; marginal area: p = 0.00000283) (Table 2). In both areas, there was an intermediate-to-strong correlation between the number of CD68-, CD163-, and CD204-positive macrophages in each examined area (intratumoral CD68- and intratumoral CD163-positive macrophages: r = 0.907; intratumoral CD68- and intratumoral CD204-positive macrophages: r = 0.876; intratumoral CD163- and CD204-positive macrophages; r = 0.86; marginal CD68- and CD163-positive macrophages: r = 0.697; marginal CD68- and CD204-macrophages: r = 0.804; marginal CD163- and CD204-positive macrophages; r = 0.72; p < 0.001 for all) (Fig. 2d–i).

### Association between the number of CD68-, CD163-, and CD204-positive macrophages in each area and clinical factors

The number of each type of marker-positive macrophage in both areas of the tumor, and their association with clinical factors, was analyzed statistically (Table 3). Sex, age, tumor size, or location had no effect on the type of marker-positive macrophage either area. However, there were significant differences among FNCLCC grades (Fig. 3). For almost all combinations of marker and area, post-hoc analysis revealed that the number of macrophages in Grade 1 cases was significantly lower than that in Grade 2 and 3 cases (Grade 1 *vs.* Grade 2 and Grade 1 *vs.* Grade 3 for intratumoral CD68-, CD163-, and CD204-positive macrophages; Grade 1 *vs.* Grade 3 for marginal CD 68-positive macrophages; Grade 1 *vs.* Grade 2 and Grade 1 *vs.* Grade 3 for marginal CD163- and CD204-positive macrophages; all p < 0.05. Grade 1 *vs.* Grade 2 for marginal CD68-positive macrophages; p = 0.09105). However, we found a significant difference between Grade 2 and Grade 3 with respect to the number of CD163-positive macrophages in the marginal area (p < 0.05). Although there was no significant difference in the number of intratumoral CD163-positive macrophages, intratumoral CD204-positive macrophages, and marginal CD204-positive macrophages between Grades 2 and 3, the overall number of macrophages in Grade 3 cases tended to be higher than that in Grade 2 cases (intratumoral CD68-positive macrophages: p = 0.4272; intratumoral CD163-positive macrophages: p = 0.08728, intratumoral CD204-positive macrophages; p = 0.5561, marginal CD68-positive macrophages; p = 0.2422, marginal CD204-positive macrophages; p = 0.1076).

### Association between the number of macrophages in the intratumoral and marginal areas and DFS

**Figure 4:**
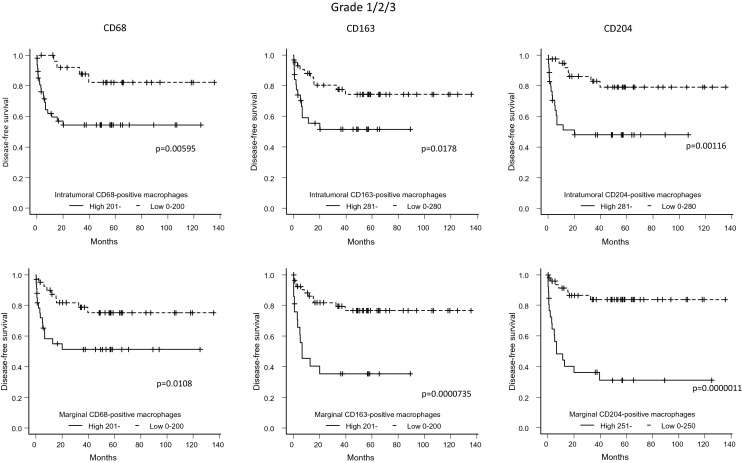
For the FNCLCC Grade 1/2/3 cases, disease-free survival (patients were grouped according to the number of marker-positive macrophages) was compared between cases with high and low macrophage numbers in the intratumoral and marginal areas (Kaplan–Meier method).

**Figure 5:**
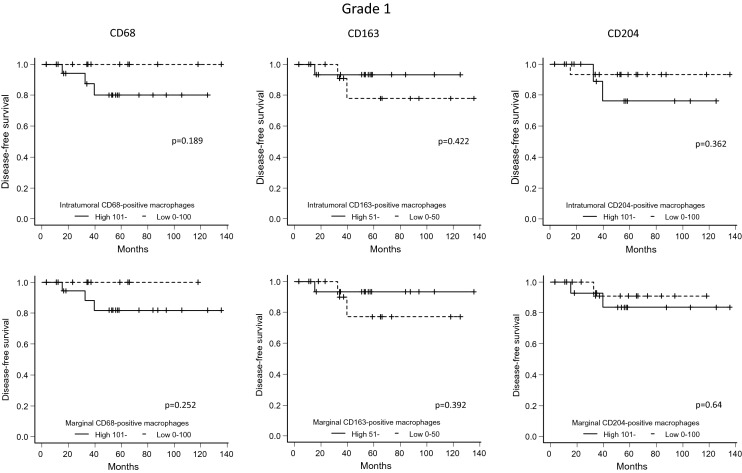
For the FNCLCC Grade 1 cases, disease-free survival (patients were grouped according to the number of marker-positive macrophages) was compared between cases with high and low numbers of macrophages in the intratumoral and marginal areas (Kaplan–Meier method).

**Figure 6:**
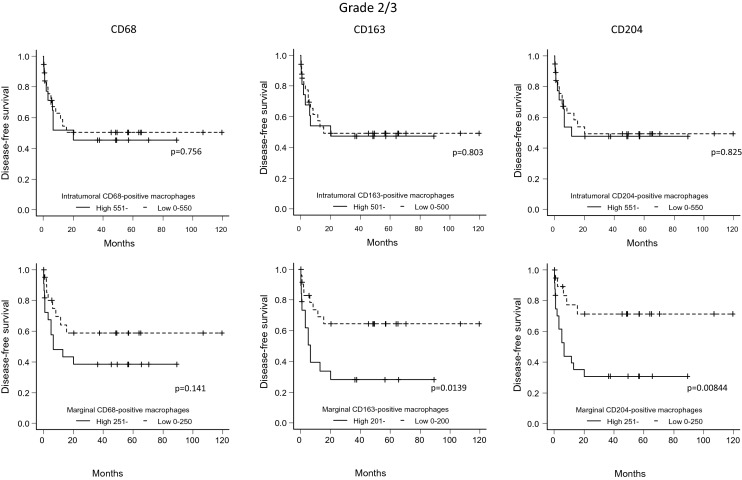
For the FNCLCC Grade 2/3 cases, disease-free survival (patients were grouped according to the number of marker-positive macrophages) was compared between cases with high and low numbers of macrophages in the intratumoral and marginal areas (Kaplan–Meier method).

**Figure 7:**
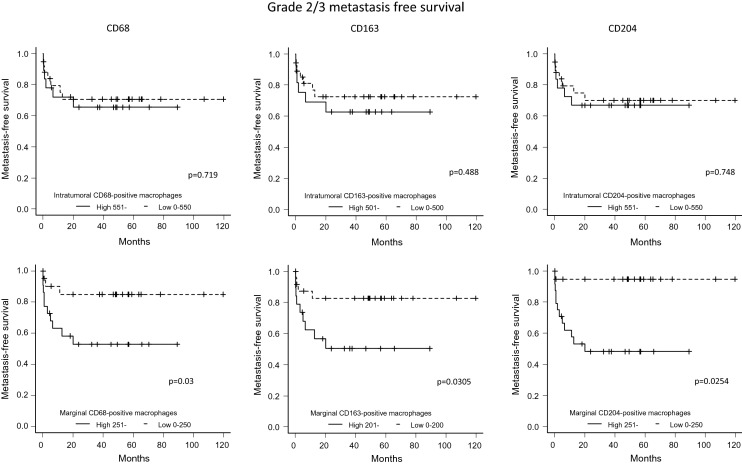
For the FNCLCC Grade 2/3 cases, metastasis-free survival (patients were grouped according to the number of marker-positive macrophages) was compared between cases with high and low numbers of macrophages in the intratumoral and marginal areas (Kaplan–Meier method).

Next, we performed a Kaplan–Meier analysis of the groups with a high and low number of CD68-, CD163-, and CD204-positive macrophages in both tumor areas. All cases with high macrophage counts in both areas, showed significantly worse DFS than those with low counts (intratumoral CD68-, CD163-, and CD204-positive macrophages: p = 0.00595, p = 0.0178, and p = 0.00116, respectively; marginal CD68-, CD163-, and CD204-positive macrophages: p = 0.0108, p = 0.0000735, and p = 0.0000011, respectively) (Fig. 4). Next, we divided the population into two subgroups (FNCLCC Grade 1 and FNCLCC Grades 2/3), and analyzed DFS. In the Grade 1 group, high numbers of CD68- and CD204-positive macrophages in both areas tended to be associated with worse DFS (intratumoral CD68- and CD204-positive macrophages: p = 0.189 and p = 0.362, respectively; marginal CD68- and CD204-positive macrophages: p = 0.252 and p = 0.64 respectively) (Fig. 5), although the results did not reach the threshold for significance. In the Grade 2/3 group, there was no significant difference between them with respect to the phenotype of macrophage in the intratumoral area; however, high numbers of CD163- and CD204-positive macrophages in the marginal area were associated with significantly worse DFS (marginal CD163- and CD204-positive macrophages: p = 0.0139 and p = 0.00844, respectively) (Fig. 6). We also assessed metastasis-free survival in patients with Grade 2/3 sarcoma (Fig. 7). High numbers of CD68-, CD163-, or CD204-positive macrophages in the marginal area were associated with poorer metastasis-free survival (marginal CD68-, CD163-, and CD204-positive macrophages: p = 0.03, p = 0.0305, and p = 0.0254, respectively); however, the number of macrophages (of any phenotype) in the intratumoral area had no significant effect on metastasis-free survival in either group. Finally, we assessed local recurrence-free survival for those with Grade 2/3 sarcoma. Again, the number and phenotype of macrophages in the intratumoral or marginal areas had no significant effect (see Supplementary Fig. S1 online).

### Prognostic value of the number of CD163- or CD204-positive macrophages in the marginal area

We performed multivariate analysis to analyze the prognostic value of the number of marginal CD163- and CD204-positive macrophages and other clinical factors (sex, age, location, size, and FNCLCC grade; see Table 4) for all cases. Multivariate analysis of DFS identified the numbers of marginal CD163- and CD204- positive macrophages as independent predictors of a poorer prognosis for Grades 1, 2, and 3 (marginal CD163- and CD204-positive macrophages: p = 0.04739 and p = 0.003541, respectively).

**Table 4:** Multivariate analysis. HRs (hazard ratios) were determined using a Cox proportional hazards model.

Marginal CD163-positive macrophages			Marginal CD204-positive macrophages		
Variable	HR(95% CI)	P value	Variable	HR (95%CI)	P value
Sex (male [n = 36] *vs*. female [n = 37])			Sex (male [n = 38] *vs.* female [n = 36])		
Male	1	0.48050	male	1	0.2533
Female	1.6330 (0.5608–3.4180)		female	1.760 (0.8534–5.145)	
Age (< 65 [n = 31] *vs.* ≥ 65 [n = 44])			Age (< 65 [n = 30] *vs.* ≥ 65 [n = 44])		
< 65	1	0.29080	< 65	1	0.1065
≥ 65	1.5800 (0.6761–3.6930)		≥ 65	2.095 (0.8861–5.473)	
Size (≤ 10 cm [n = 38] *vs.* > 10 cm [n = 37])			Size (≤ 10 cm [n = 38] *vs*. > 10 cm [n = 36])		
≤ 10 cm	1	0.29490	≤ 10 cm	1	0.3306
> 10 cm	1.6330 (0.6523–4.0870)		> 10 cm	1.578 (0.6295–3.955)	
Location (extremities [n = 52] *vs*. others [n = 23])			location (extremities [n = 51] *vs*. others [n = 23])		
Extremities	1	0.52140	Extremities	1	0.4378
Others	1.3410 (0.5472–3.2850)		others	1.417(0.5875–3.417)	
Grade (grade 1 [n = 31] *vs*. Grade 2/3 [n = 44])			Grade (grade 1 [n = 31] *vs*. Grade 2/3 [n = 43])		
Grade1	1	0.01816	Grade1	1	0.06349
Grade2/3	4.7060 (1.3020–17.0100)		Grade2/3	3.509 (0.9319–13.210)	
Marginal CD163 + macrophages (high [n = 54] *vs*. low [n = 21])			Marginal CD204 + macrophages (high [n = 50] *vs.* low [n = 25])		
Low (0–200)	1	0.04739	low (0–250)	1	0.003541
High (≥ 201)	2.542 (1.0110–6.394)		high (≥ 251)	4.694 (1.6600–13.270)	

## Discussion

Compared with cancer in general, sarcoma is quite rare. Furthermore, soft-tissue sarcoma includes many histological types; therefore, it is difficult to collect cases limited to a specific single histological type for statistical analysis. For these reasons, few studies have performed clinicopathological examination of TAMs in sarcoma. Here, we also reviewed previous studies that investigated the association between TAMs and clinical outcomes of patients with soft-tissue sarcoma (Table 5)^7,30–34^. Most previous studies report that high macrophage counts are associated with poor outcomes; however, some do not show a significant association between TAMs and clinical outcome. Also, none of these studies evaluated macrophages in the marginal area, as we did here.

**Table 5:** Review of the literature regarding the association between TAMs in sarcoma and clinical outcome.

Author	Number of cases	Examined factors	Results
Chan-Han Lee et al. ^28^	149 LMS cases	Patients categorized into 3 groups according to the number of CD68 + and CD163 + macrophages in a 0.6 mm tumor core	High density of CD68 + and CD163 + was correlated with poor disease specific survival in nongynecological leiomyosarcomas
Kristen et al. ^29^	52 LMS cases	Number of CD163 + macrophages. Expression of CD163, CD16, and cathepsin L was evaluated as “colony stimulating factor-associated protein”	Increased levels of CD163 + tended to decrease OS, but the difference was not statistically significant. High expression of CD16, cathepsin L, and all (CD16, CD163, cathepsin L) correlated with worse OS of patients with gynecological leiomyosarcoma
Nabesima et al.^30^	78 myxoid liposarcoma cases	Number of CD68 + and CD163 + macrophages in 10 random high-power fields	High levels of CD68 + and CD163 + were associated with poorer overall survival
Oike et al.^31^	36 synovial sarcoma cases	Lymphocytes (CD4 + , CD8 + , and FOXP3 +) and macrophages (CD163 +) were counted in five high-power fields. Expression of PD-L1 by sarcoma cells was examined	High numbers of CD163 + macrophages were associated with significantly worse OS and progression-free survival
Shiraishi et al.^32^	62 UPS cases	The number of Iba1 + and CD163 + macrophages in 10 randomly selected high-power fields was counted	A high percentage of CD163 + macrophages (CD163 + /Iba1 +) was associated with poorer OS
Komohara et al.^4^	28 UPS cases	The number of macrophages (Iba1 + , CD163 + , and CD204 +) and CD8 + lymphocytes in five randomly selected high-power views was counted	A high number of CD163 + and CD204 + macrophages tended to indicate worse DFS and OS, but result was not significant

LMS, leiomyosarcoma; UPS, undifferentiated pleomorphic sarcoma.

We collected cases regardless of histology; however, we excluded cases that had undergone preoperative chemotherapy or radiotherapy. In the overall population, the macrophage count (all phenotypes) in both areas showed a significant association with DFS. These results suggest that the number of macrophages in the intratumoral or marginal area is associated with the clinical grade of sarcoma. This is supported by our finding that the number of macrophages, especially CD163- or CD204-positive macrophages, in the intratumoral or marginal area was associated with FNCLCC grade (Fig. 3). In the Grade 1 subgroup, high numbers of CD68- and CD204-positive macrophages tended to be associated with a poorer prognosis, although there was no significant difference between macrophage numbers in the two areas. We think that if the number of cases had been larger, we may have found a significant difference. Interestingly, we found that patients in the Grade 2/3 subgroup with high numbers of CD163- and CD204-positive macrophages in the marginal area showed significantly poorer DFS; this association was not present when we examined the intratumoral area. The finding that high numbers of intratumoral macrophages (CD68-positive, CD163-positive, or CD204-positive) tended to be associated with somewhat poorer DFS is consistent with previous studies. To evaluate DFS, we defined local recurrence and metastasis as a clinical event. We considered that local recurrence depends somewhat upon the surgical method (marginal or extensive resection); thus, we also assessed metastasis-free survival in those with Grade 2/3 sarcoma. We found that those with high numbers of macrophages in the marginal area showed poorer outcomes (as was observed for DFS). Multivariate analysis identified a high number of CD163- or CD204-positive macrophages in the marginal area as an independent prognostic factor.

We suggest three explanations for the above results. One involves the types of cases that were enrolled in the study. Unlike previous studies, our cases were of various histological type. The pathological characteristics and clinical behavior of sarcomas differ among histological types, which may be one reason for the differences in the results. Second, the accuracy of the macrophage counts should be considered. In cases of high-grade sarcoma, especially undifferentiated sarcoma, the sarcoma cells themselves are sometimes CD68-positive. Actually, almost all cases diagnosed as undifferentiated pleomorphic sarcoma today may have been previously diagnosed as malignant fibrous histiocytoma. We did not find an sarcoma cells that were CD163- and CD204-positive; however, the possibility that some were CD163- or CD204-positive cannot be ruled out (indeed, a case of CD163-positive sarcoma has been reported^35^). In addition, high-grade sarcomas often show dense infiltration of the intratumoral area by CD68-/CD163-/CD204-positive cells, as demonstrated in Fig. 1c. These findings make precise counting of CD68-, CD163-, and CD204-positive cells very difficult; thus, the number of macrophages in the intratumoral area may not be precise. In turn, the number of macrophages in the marginal area, which was lower than that in the intratumoral area, was easier to count because the macrophages were easier to distinguish from sarcoma cells. Of course, quantification of the macrophages in the marginal area was also associated with some problems. One such problem occurs in cases involving marginal resection. Some cases, especially low-grade sarcoma (e.g., atypical lipomatous tumors) are treated by marginal resection. In this study, we carefully analyzed small amounts of the non-sarcoma (i.e., normal) area adjacent to the sarcoma and counted the number of macrophages in those areas. However, in cases in which we could not find a marginal normal area, the marginal tumor area was investigated instead. Another problem is that there were cases with an unclear demarcation line. In such cases, we carefully chose the area to analyze, making sure not to count sarcoma cells. Despite these problems, for Grade 2/3 sarcomas, the number of the macrophages in the marginal area show a stronger correlation with prognosis than the number in the intratumoral area. Lastly, marginal macrophages may play an important role in sarcoma progression. As we described earlier, macrophages play an important role in formation of the tumor microenvironment in gastric cancer^28^. If this is the same for sarcoma, then the number of macrophages in the marginal area may reflect the clinicopathological behavior of the sarcoma.

Biomolecular studies are needed to confirm the hypothesis that macrophages in the marginal area play an important role in progression of sarcoma. Similar to other studies, the results of the present study suggest that there are therapeutic benefits in targeting macrophages as a treatment for sarcoma^36^.

## Conclusion

We investigated the numbers of CD68-, CD163-, and CD204-positive macrophages in the intratumoral and marginal areas of sarcoma cases. In all cases (Grade 1/2/3), we found that patients with high numbers of intratumoral macrophages (CD68 + , CD163 + , and CD204 +) and high numbers of marginal macrophages (CD68 + , CD163 + , and CD204 +) showed poor DFS. In Grade 2/3 cases, those with high numbers of CD163- and CD204-positive macrophages in the marginal area showed a poorer prognosis than those with low numbers, whereas there was no significant difference in prognosis of those with high and low numbers of CD68-, CD163-, or CD204-positive macrophages in the intratumoral area.

## Supplementary Information

Supplementary Information.

## Data Availability

All data generated or analyzed during this study are included in the published article.
