# Supplementary material for: Macrophage numbers in the marginal area of sarcomas predict clinical prognosis
Source: Sci Rep. 2023 Jan 23;13:1290. doi: 10.1038/s41598-023-28024-1 (PMC9870999; doi:10.1038/s41598-023-28024-1)
Supplement: Supplementary file 1 — Supplementary Information. [file 41598_2023_28024_MOESM1_ESM.pptx]

## Slide 1
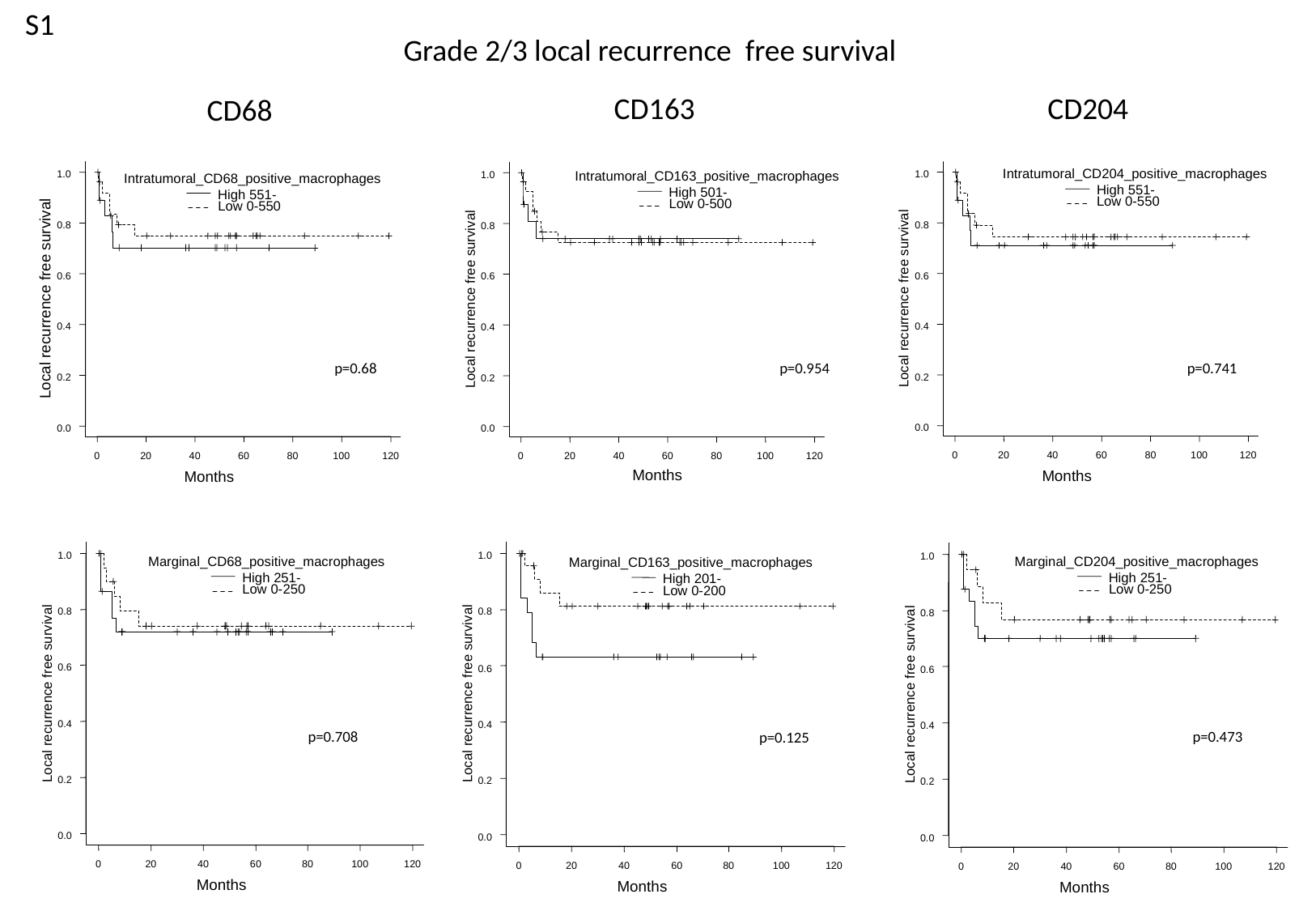

S1
Grade 2/3 local recurrence free survival
CD163
CD204
CD68
1.0
0.8
0.6
Local recurrence free survival
0.4
0.2
0.0
0
20
40
60
80
100
120
Months
1.0
0.8
0.6
Local recurrence free survival
0.4
0.2
0.0
0
20
40
60
80
100
120
Months
Intratumoral_CD163_positive_macrophages
1.0
High 501-
Low 0-500
0.8
0.6
Local recurrence free survival
0.4
0.2
0.0
0
20
40
60
80
100
120
Months
Intratumoral_CD204_positive_macrophages
Intratumoral_CD68_positive_macrophages
High 551-
High 551-
Low 0-550
Low 0-550
p=0.68
p=0.954
p=0.741
1.0
0.8
0.6
Local recurrence free survival
0.4
0.2
0.0
0
20
40
60
80
100
120
Months
1.0
0.8
0.6
Local recurrence free survival
0.4
0.2
0.0
0
20
40
60
80
100
120
Months
1.0
0.8
0.6
Local recurrence free survival
0.4
0.2
0.0
0
20
40
60
80
100
120
Months
Marginal_CD204_positive_macrophages
Marginal_CD68_positive_macrophages
Marginal_CD163_positive_macrophages
High 251-
High 251-
High 201-
Low 0-250
Low 0-250
Low 0-200
p=0.708
p=0.473
p=0.125
